# Induction of Neural Progenitor-Like Cells from Human Fibroblasts via a Genetic Material-Free Approach

**DOI:** 10.1371/journal.pone.0135479

**Published:** 2015-08-12

**Authors:** Fahimeh Mirakhori, Bahman Zeynali, Hassan Rassouli, Ebrahim Shahbazi, Shiva Hashemizadeh, Sahar Kiani, Ghasem Hosseini Salekdeh, Hossein Baharvand

**Affiliations:** 1 School of Biology, College of Science, University of Tehran, Tehran, Iran; 2 Department of Stem cells and Developmental Biology at Cell Science Research Center, Royan Institute for Stem Cell Biology and Technology, ACECR, Tehran, Iran; 3 Department of Molecular Systems Biology at Cell Science Research Center, Royan Institute for Stem Cell Biology and Technology, ACECR, Tehran, Iran; 4 Department of Developmental Biology, University of Science and Culture, ACECR, Tehran, Iran; University of Nebraska Medical Center, UNITED STATES

## Abstract

**Background:**

A number of studies generated induced neural progenitor cells (iNPCs) from human fibroblasts by viral delivering defined transcription factors. However, the potential risks associated with gene delivery systems have limited their clinical use. We propose it would be safer to induce neural progenitor-like cells from human adult fibroblasts via a direct non-genetic alternative approach.

**Methodology/Principal Findings:**

Here, we have reported that seven rounds of TAT-SOX2 protein transduction in a defined chemical cocktail under a 3D sphere culture gradually morphed fibroblasts into neuroepithelial-like colonies. We were able to expand these cells for up to 20 passages. These cells could give rise to cells that expressed neurons and glia cell markers both *in vitro* and *in vivo*.

**Conclusions/Significance:**

These results show that our approach is beneficial for the genetic material-free generation of iNPCs from human fibroblasts where small chemical molecules can provide a valuable, viable strategy to boost and improve induction in a 3D sphere culture.

## Introduction

The unique capability of neural progenitor cells (NPCs) to induce regeneration in several animal models of neurological disorders [1] make them the best potential cell source for regenerative medicine. However, the low availability of reliable human NPC sources limits their validity for these treatments. The ideal cell source for cell-based therapies and their downstream use in drug discovery and regenerative medicine should be patient specific, easy to obtain and expand with controlled differentiation, have the capability to generate desired derivatives, as well as safety and non-tumorigenicity [2]. Human NPCs can be generated from pluripotent stem cells (PSCs), however the remainder of even a few undifferentiated PSCs in the resultant cell mixture can cause tumor formation after transplantation [3,4]. To address this problem, tremendous efforts have been undertaken to convert one cell type directly into induced neural progenitor cells (iNPCs) from accessible cell types such as skin fibroblasts by forced expression of defined transcription factors [5–9].

This modern-day alchemy also represents a useful tool for basic research, drug screening, disease modeling and therapeutic discovery because they have the ability to produce a sufficient amount of cells for downstream applications [10–12]. Currently there are numerous ways in which iNPCs can be obtained from various differentiated somatic cell types [13–19]. The generation of multipotent NPCs from mouse and human fibroblasts by overexpression of *SOX2* alone provides an opportunity to obtain an ideal NPC source for human therapy [9]. However, because of the use of exogenous genes major clinical safety issues still remain to be overcome such as the potential risks associated with the use of viruses, genetic material transfection, the risk of mutagenesis and unpredictable genetic dysfunction [20].

An attractive approach to solve these safety issues involves the use of proteins of desired keystone genes that contain a protein transduction domain of the human immunodeficiency virus transactivator of transcription (HIV/TAT) [21]. Researchers have utilized TAT ability to translocate various biomolecule cargoes (such as drug molecules, nucleic acids and particularly large proteins), as well as its ability to cross the plasma and nuclear membrane and trigger expected cell responses to reprogram mouse and human fibroblasts in the absence of genetic intervention [22–25]. Several studies reported that specific culture conditions can evoke cellular reprogramming and transdifferentiation [15,26–28]. Recent studies have reported that a chemical cocktail and specific culture condition [28,29] could also induce mouse somatic cells to iNPCs without introducing exogenous factors by activation of endogenous *SOX2* expression. The advantage of conversion by these systems over viral gene delivery is the applicability to all cell types, controlled administration, and generation of genetic material-free cell sources.

In this study, we have sought to determine whether human fibroblasts could be induced to NPCs via a genetic material-free approach. Here, we generated human iNPCs without any genetic alterations by using SOX2 in the form of a TAT fusion protein and the presence of a chemical cocktail under 3D sphere culture conditions.

## Materials and Methods

### TAT recombinant proteins production

The pDest17/TAT-SOX2 and pDest17/TAT-EGFP constructed by Gateway Technology (Invitrogen, Carlsbad, CA, USA). Expression clones were transferred into Library Efficiency DH5α Competent Cells (Invitrogen, Carlsbad, CA, USA) by the heat shock method as described by the manufacturer for confirming recombination and making glycerol stock for further uses. Confirmed clones were used for recombinant protein production.

Recombinant protein production was performed as previously described [30]. Briefly, pDest17/TAT-SOX2 and TAT-EGFP expression vector were transformed into *E*. *coli* strain BL21 competent cells (DE3; Novagen,WI, US). The transformed cells were cultured to reach an OD 600 ~ 0.8 and then induced by 1 mM isopropyl-beta-D-thiogalactopyranoside (IPTG, Fermentas, Lithuania). His6 was also used for protein purification. The His6-TAT-SOX2 and His6-TAT-EGFP proteins were purified by the Ni-NTA Fast Start Kit (Qiagen, USA) in denature condition. Immobilized SOX2 proteins eluted with 8 M urea (pH 3.5), then desalted by Tris (5 mM) that contained 50% glycerol and maintained at -20°C until use. The purified proteins were analyzed by SDS-PAGE, Coomassie blue staining (S1 Fig).

### Cell culture and iNPCs generation

Human foreskin fibroblasts were kindly provided by the Royan Institute Stem Cell Bank (Iran) as a primary cell culture (male donor, 7-day old). This study was approved by the ethical committee of Royan Institute while the written consent of the donor's parent was obtained. The human fibroblasts were maintained in fibroblast medium (FM: DMEM, Invitrogen) supplemented with 10% fetal bovine serum (FBS, Invitrogen). For the 3D sphere culture, initial cells were seeded at a density of 1 × 10^6^ cells/mL onto agarose coated plates [29]. After 48 h in FM, the medium replaced by M1 [DMEM/F12; neurobasal (1:1) supplemented with 7.5% KSR, 2.5% FBS, 1% N2, 1% B27 (all from Invitrogen)]; 10 ng/ml human LIF (Royan Institute); and a cocktail of small molecules (SM) composed of 5 nM LDN, 10 μM SB431542, 3 μM CHIR99021, 2 μM purmorphamine, and 50 μM VPA (all from Sigma-Aldrich). The medium was replenished every other day and protein transductions were carried out in seven repeated transduction cycles (every 48 h) using the TAT-SOX2 protein (Royan Institute) with a total protein amount of 10 μg/ml per transduction cycle. After 14 days of protein transduction, cells were dissociated with trypsin-EDTA (Invitrogen) and then reseeded onto Matrigel (Invitrogen) pre-coated plates (Nunc) in M2:NPC medium [DMEM/F12: neurobasal 1:1, N2, and B27 supplemented with 20 ng/ml bFGF [30] and EGF] supplemented with SM (2 μM SB431542, 1 μM CHIR99021, and 0.5 μM purmorphamine). The same medium was used for cell expansion. The medium was changed every two days.

### *In vitro* adherent colony formation assay

Fully dissociated cells were seeded onto laminin-coated 96-well plates (Nunc) at an average density of 1 cell per well and cultured in our M2 medium. All wells were visually inspected under phase contrast microscope (Zeiss) to exclude wells that contained no cells or more than one cell. After a two-week culture period, the wells were observed for the presence of sphere colonies. Next, the primary clones were dissociated into single cells which were cultured as one cell/well in 96-well culture plates with the intent to generate sub-clones (secondary spheres).

### *In vitro* differentiation

Dissociated cells were reseeded onto laminin coated plates in differentiation medium [Neurobasal with 1% N2, 2% B27, 1% NEAA supplemented with 10 ng/ml BDNF, 10 ng/ml GDNF, and 200 μM ascorbic acid (all from Sigma-Aldrich), without growth factors- bFGF and EGF] for up to 3–4 weeks and differentiated spontaneously. Half the volume of the medium was replaced by fresh medium every 2–3 days. For spheroid differentiation, cells were trypsinized into single cells and transferred into non-adherent plates in EB medium without any anti-differentiation factors.

### Real time-PCR

RNA extraction was performed using the TRIzol reagent according to the manufacturer’s instructions (Sigma-Aldrich). Extracted RNA was used for cDNA synthesis with a Reverse Transcription kit (Takara). Quantitative RT-PCR (qRT-PCR) was performed by using the SYBR Green kit (Takara). All primer sequences used in this study are listed in S1 Table. We used the 2^(-Delta Delta CT)^ method and the normalizer gene for our studies was *GAPDH*. At least three independent samples were analyzed.

### Immunocytochemistry and flow cytometry

Cells were fixed in 4% neutral-buffered paraformaldehyde (PFA) for 20 min at 4°C and permeabilized with 0.05% triton X-100 in phosphate-buffered saline (PBS) for 10 min at room temperature, then blocked with 10% secondary host serum in 1% bovine serum albumin in PBS (BSA-PBS) for 1 h at room temperature. These cells were subsequently incubated overnight at 4°C with primary antibodies diluted in PBS that contained 1% BSA. The cells were washed three times with PBS plus 0.02% Tween 20 and incubated with their respective secondary antibodies conjugated to Alexa Fluor 568 and 546 (red) or Alexa Fluor 488 (green) in the dark for 30 min at room temperature. The cells were washed three times and then counter stained with 4, 6-diamidino-2-phenylindole (DAPI). Finally cells were observed using a fluorescence microscope (Zeiss). For quantitation, the number of positive cells was scaled to the total cells based on DAPI staining of the nuclei.

For flow cytometry, cells were harvested with 0.25% trypsin/EDTA, centrifuged, resuspended, and fixed in 4% PFA at room temperature for 10 min. Then cells (1–5 × 10^5^) were permeabilized, blocked and incubated with primary antibodies or the isotype control antibody diluted in PBS that contained 1% BSA as described above. After washing cells with washing buffer, the cells were incubated with secondary antibodies in the dark for 30 min at room temperature, washed with washing buffer, and resuspended in PBS. Flow cytometry was performed using a FACScalibur (BD Biosciences) and the data were analyzed by software version 2.5. All primary and secondary antibodies, sources, and dilutions are listed in S2 Table.

### Transplantation of iNPCs, tissue processing and immunofluorescence staining

Royan Institutional Review Board and the Institutional Ethical Committee approved all study experiments. Animals were maintained according to the guidelines for laboratory animal care and safety. The approval ID is J/90/1397. In order to generate green fluorescent protein (GFP)-expressing iNPCs, we infected cells with lenti-GFP virus (Gene art). 2-day-old rat Pups were cryoanesthetized in melting ice. Fluorescent protein (GFP)-expressing iNPCs were suspended at 10^5^ cells/μl in PBS. Each rat received 2 μl iNPCs injection slowly into the both right and left hemispheres at 1 mm from the midline and 1 mm below the surface of skull using Hamilton syringe. After 10 days of cell transplantation following anesthesia, the brains were collected and post fixed in 4% paraformaldehyde, and then transferred in 20% sucrose solutions. Coronal sections (10 μm) were cut on a cryostat. The immunohistochemisty was performed as described by Pouya et al. [31]. All antibodies, sources, and dilutions are listed in S2 Table.

### Statistical analysis

Values were expressed as mean ± SD. Differences between means were assessed by the *t*-test. A p-value of <0.05 was considered statistically significant.

## Results

### Assessment of TAT recombinant protein transduction

The efficacy of transduction is affected by protein concentration [32], therefore we treated the human fibroblasts with 5, 10 and 20 μg/ml TAT recombinant proteins. We observed that the optimal concentration was between 10–20 μg/ml but higher concentrations resulted in excessive cell death (data not shown). We chose 10 μg/ml for our following experiments in order to minimize any potential cytotoxic effects.

**Fig 1:**
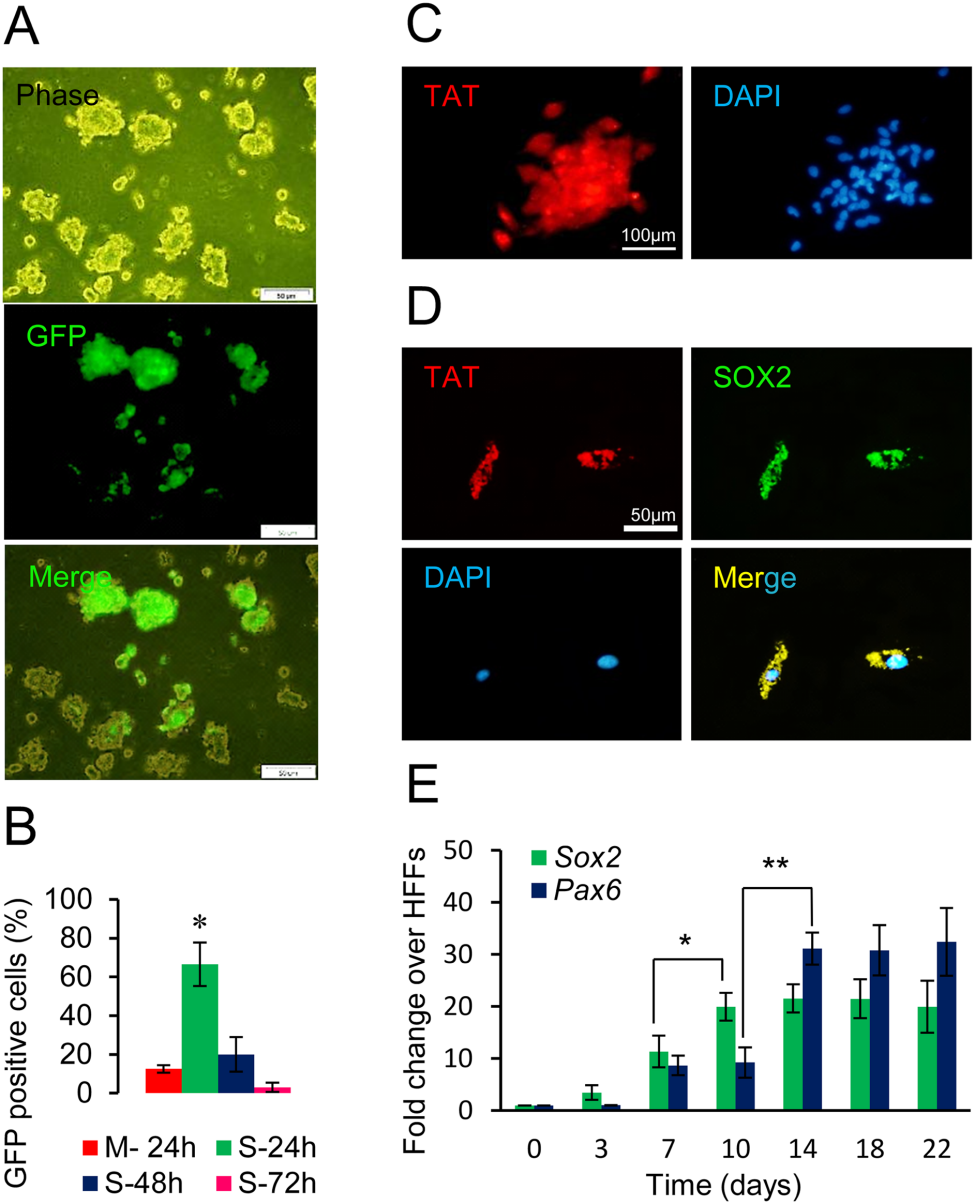
Assessment of TAT recombinant protein transduction. (A) Visualization of fusion proteins in in suspension cultured HFFs transduced with 10 μg/ml TAT-EGFP for 24 h. Scale bars represent 200 μM. (B) To determine protein stability, 10 μg/ml of TAT-EGFP proteins were added to HFFs for 24, 48 and 72 h, and the presence of transduced protein in the cells were analyzed by flow cytometery (M; monolayer and S; 3D sphere culture). (C) The uptake and stability of the TAT fusion protein were visualized by TAT immunostaining of transduced human fibroblasts after 24 and 48 h post-TAT-SOX2 transduction (Scale bars: 200 μm). (D) The middle panels represent double staining TAT and SOX2 with higher magnification in transduced cells at 24 h post-transduction (50 μm). The nuclei were counterstained with DAPI. (E) Determining the time required for protein transduction. Relative expression of neural markers (*SOX2* and *PAX6*) induced by 10 μg/ml TAT-SOX2 with a 48 h transduction interval (d0: Non-transduced cells).

We assessed the influence of the 3D sphere culture which supported the protein/cell interaction to promote intracellular delivery. Fibroblasts are routinely expanded as a monolayer on conventional tissue cultures. When human fibroblasts were grown in a 3D sphere culture under low attachment conditions, they formed spheres with a mean size of 78 ± 56 μm (n = 100) within 24 h (Fig 1A). This 3D sphere culture system might induce reprogramming signals towards neural stem cells in human fibroblasts [28,29,33]. We could not begin protein transduction on the day of the initial cell seeding (-2d), due to an excessive cell death and non-compact cell aggregates. Therefore, we decided to treat cells 48 h after initial cell seeding (day 0) which resulted in aggregate formation and cell adaptation. In order to visualize the ability of the recombinant TAT to enter its cargo proteins into the cultured fibroblasts under 3D sphere culture, we used a TAT-EGFP fusion protein as a control recombinant protein. We demonstrated that TAT could efficiently translocate EGFP into the fibroblasts within 4–8 h post-transduction (data not shown). After 24 h, many spheres were EGFP positive however there were a few positive cells in the monolayer culture (Fig 1A and S2A Fig). We observed that TAT harboring proteins efficiently transduced cells in a 3D structure (Fig 1B). The delivered proteins degraded gradually and were absent 48–72 h post-transduction. To estimate the efficiency of the protein transduction and the time course in which the proteins could subsist inside the cells, we used flow cytometry to analyze the TAT-EGFP fusion proteins present in these cells at 24, 48, and 72 h post-transduction. The results showed that at 24, 48, and 72 h post-transduction, 66%, 40%, and 3% of the transduced cells, respectively, were fluorescent, whereas in monolayer was very lower than these levels (Fig 1B, and S2A Fig). Thus the 3D sphere culture was better condition for protein transduction since the relative amount of transduced cells was increased and the relative stability of TAT fusion proteins was higher than that of monolayer-around 48–72 h post-transduction; under monolayer culture conditions this stability was less than 24 h (Fig 1B, S2A and S2B Fig). These results demonstrated that TAT was capable of delivering EGFP as an active protein into human fibroblasts under 3D sphere culture conditions.

We employed the recombinant SOX2 protein to address whether it was possible to generate iNPCs from human fibroblasts without DNA vectors by TAT-mediated protein transduction. The recombinant TAT-fusion proteins were synthesized by utilizing a bacterial expression vector. Cellular uptake of the TAT-SOX2 protein was visualized by probing for the presence of recombinant protein. Immunocytochemistry demonstrated that the proteins appeared to be sustained inside the cells for up to 24 h as the majority of cells stained positive (Fig 1C). TAT-SOX2 protein entered the cells where the fusion protein was localized in ‘endosome-like’ vesicles in the cytoplasm, around and inside the nucleus (Fig 1D). However, the percent of positive cells decreased over time (Fig 1B and S2C Fig). Unlike viral- or other DNA-based methods, recombinant proteins must be continually supplied and repeat cycles were necessary for successful transduction. A time-dependent uptake was observed following protein transduction, which indicated that the frequency of treatment must be every 48 h (Fig 1C and 1D). We tested the effects of 1 to 12 treatment cycles with a 48 h transduction interval on the expression of neural markers (*SOX2* and *PAX6*) that were human neuroectoderm cell fate determinants (Fig 1E). When this procedure was repeated for further cycles, we observed excessive cell death (data not shown). Because the expression of *SOX2* and *PAX6* showed no significant differences between days 10–18 and 14–18 respectively, we chose day 14 with 7 rounds of protein treatment. Our results showed that SOX2 recombinant proteins could be delivered intracellularly as biologically active proteins that had the capability to induce neural gene expression in human fibroblasts. Therefore, we used this system as a tool to deliver our target protein (SOX2) into the human fibroblasts, which triggered the neural conversion events, as observed by the endogenous expression of *PAX6* and *SOX2*.

### Generation of iNPCs from human fibroblasts

As previously mentioned, in order to generate iNPCs from human fibroblasts we initially cultured the cells in a 3D sphere culture. Cells were subsequently subjected to seven repeated protein transduction cycles at 48 h intervals. After completion of the protein transduction cycles, the cells were finely dissociated into single cells and transferred onto Matrigel-coated plates in NPC medium to achieve an adherent culture (S3 Fig). With this process, the expression of NPC markers (*SOX2*, *PAX6*, *NESTIN*, and *OLIG2*) were up regulated (S3A Fig). We demonstrated that the 3D sphere culture alone (-SOX2/-SM) markedly induced *OLIG2* and *NESTIN* expressions in human fibroblasts, and lower extend *SOX2* and *PAX6*. However, protein transduction could substantially increase the *SOX2* and *PAX6* levels (+SOX2/-SM S3A Fig). Furthermore, the treated cells acquired different behaviors and morphologies compared to their initial human fibroblasts (S3B Fig). However, no colonies formed after further incubation on NPC expansion medium (S3B Fig) and after a number of passages the culture contained a high incidence of dying cells. Interestingly, when the medium was changed to differentiation medium very few cells exhibited immature neuronal morphology. We therefore concluded that these cells should not be considered as an expandable stem cell line. This might be due to the fact that the transduced cells were partially converted and could not be maintained under this condition (S3A Fig).

**Fig 2:**
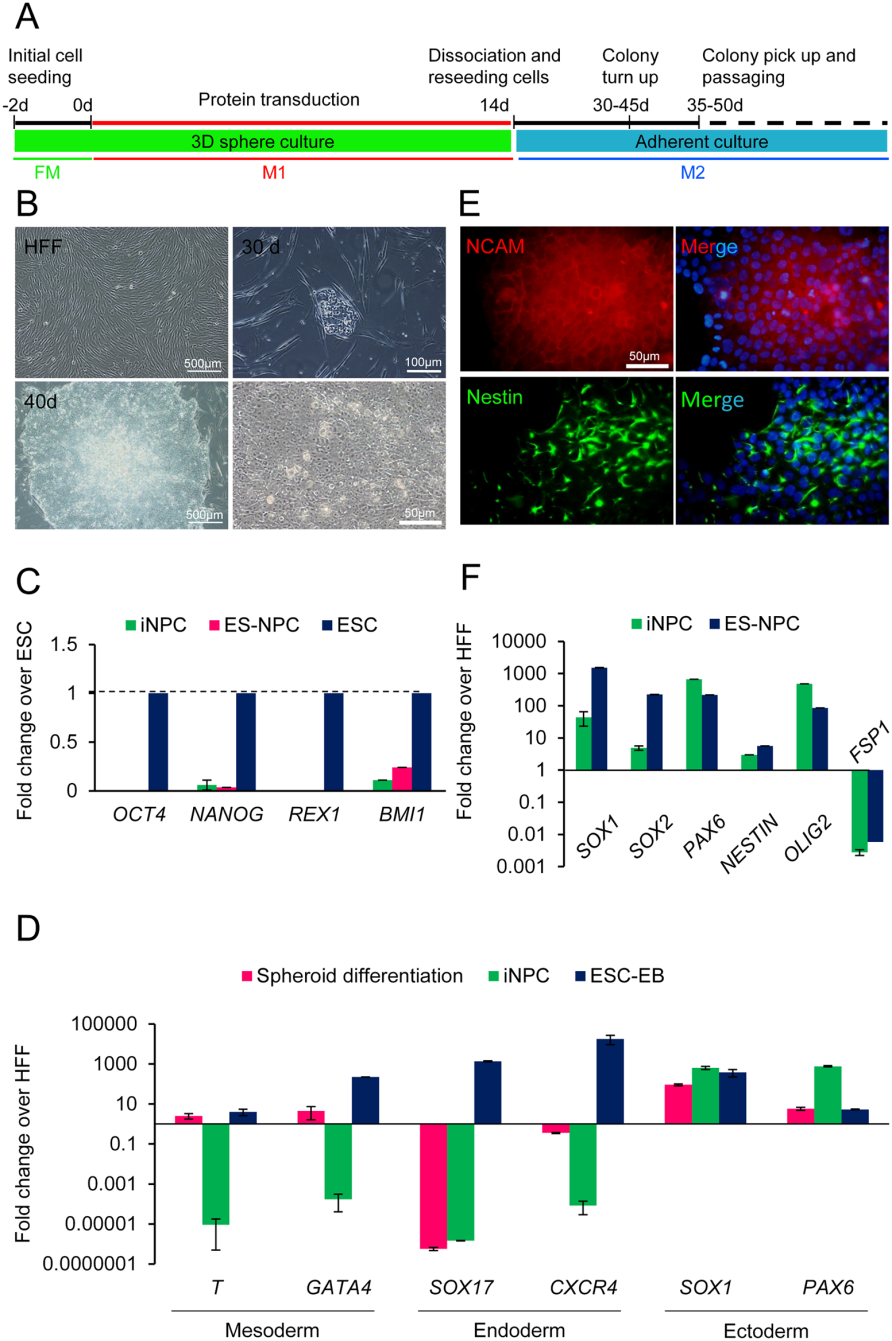
Generation and characterization of protein iNPCs from human fibroblasts. (A) Schematic representation of the protein iNSCs generation strategy. (B) Morphological changes of human fibroblasts through iNPCs generation. Phase-contrast image of human fibroblasts (left panel) and an early iNPC colony generated by a combination of small molecule (SM) and TAT-SOX2 protein transduction (right). The lower pictures represent the shape of a large induced colony after 40 days and morphological changes of the cells in the induced colonies (right). (C) Quantitative-PCR analysis of pluripotency markers (*Oct4*, *Nanog*, *Rex1* and *Bmi1*). (D) Real-time RT-PCR analysis of iNPCs in NSC expansion medium (M2) and embryoid body (EB)-like conditions (spheroid differentiation) for neural and non-neural lineages. All sample data (d, e and f) are normalized to that of non-treated human fibroblasts, which is considered as 1. (E) The induced colonies were NCAM, and Nestin positive. The nuclei were counterstained with DAPI. (F) Real-time analysis of neural progenitor markers: *SOX1*, *SOX2*, *PAX6*, *Nestin*, and *OLIG2*. ES-derived neural stem cells (ES-NPCs) were used as the positive control.

We were therefore interested in investigating the completion of this conversion and induce NPC-like colonies using chemicals. We tested a condition that could promote NPC maintenance, SOX2 expression, and facilitate neural conversion efficiency [15,28]. We treated cells in NPC medium supplemented with recombinant human LIF and a chemical cocktail composed of SB431542 (TGF-β inhibitor), CHIR99021 (GSK3b inhibitor) [35], valproic acid (VPA- an HDAC inhibitor that can significantly improve reprogramming and neural conversion efficiency) [23,28] and purmorphamine (hedgehog signaling agonist, [26])–all referred to M1, for the first 14 days (Fig 2A). Then, cells were transferred onto Matrigel-coated plates to establish an adherent culture and, at the same time, we replaced the medium with M2 medium. The cell culture process is also outlined in Fig 2A.

Under this modified condition (+SOX2/+SM), small colonies were observed at approximately 30 to 40 days (~35±8 days in different independent experiments) post-protein transduction (Fig 2B). We also obtained 2.2 ± 0.48 colonies per 5 × 10^6^ cells which called them iNPCs. In contrast, no such colonies were observed in either those only treated with SM (-SOX2/+SM or SOX2 (+SOX2/-SM); S3B Fig), even though NPC related genes upregulated at the early stage of the procedure (S3A Fig).

The first induced colony emerged as a small cluster which grew rapidly over the following days; by days 35–40 these colonies were large enough to be manually picked up (Fig 2B). The colony had an epithelial-like morphology. The cells showed a small round morphology with a large clear nucleus and two to three nucleoli (a high nucleus to cytoplasm ratio; Fig 2B). We hypothesized that these colonies might go through a pluripotent state. To address this assumption, we assessed the expressions of pluripotency markers (*OCT4*, *NANOG*, *REX1*, and *BMI1*). These markers were trivial in iNPCs compared to human ES cells, but they were more similar to ES-NPCs (Fig 2C). We analyzed the expression levels of three primary germ layer marker genes in our expansion medium (M2) and in an embryoid body (EB)-like condition or spheroid differentiation to make sure the generated cells are not pluripotent. Real-time PCR analysis revealed the expression of neuroectodermal related genes (*SOX1*, *PAX6*) whereas non-neural lineage markers, Brachyury (*T*), *SOX17*, *GATA4*, and *FSP1* were repressed (Fig 2D).

We used immunofluorescence staining to determine if these colonies had neural stem/progenitor-like characteristics by examining them for the presence of neuronal markers. The emerging neuroepithelial-like colonies were confirmed by staining of neural stem cell markers NCAM and NESTIN (Fig 2E). Untransfected human fibroblasts were negative for NPC related markers (S4 Fig). Quantitative real-time RT-PCR (qRT-PCR) confirmed the expressions of NPC marker genes including *SOX1*, *SOX2*, *PAX6*, *NESTIN*, and *OLIG2* (Fig 2F).

These data suggested a direct conversion nature of our NPC-like colonies as the related gene expression levels were distinct from ES cells and non-neural lineage populations in addition to an upregulation of neural genes *SOX1*, *SOX2*, *PAX6*, and *NESTIN*.

### Characterization of iNPCs from human fibroblasts

**Fig 3:**
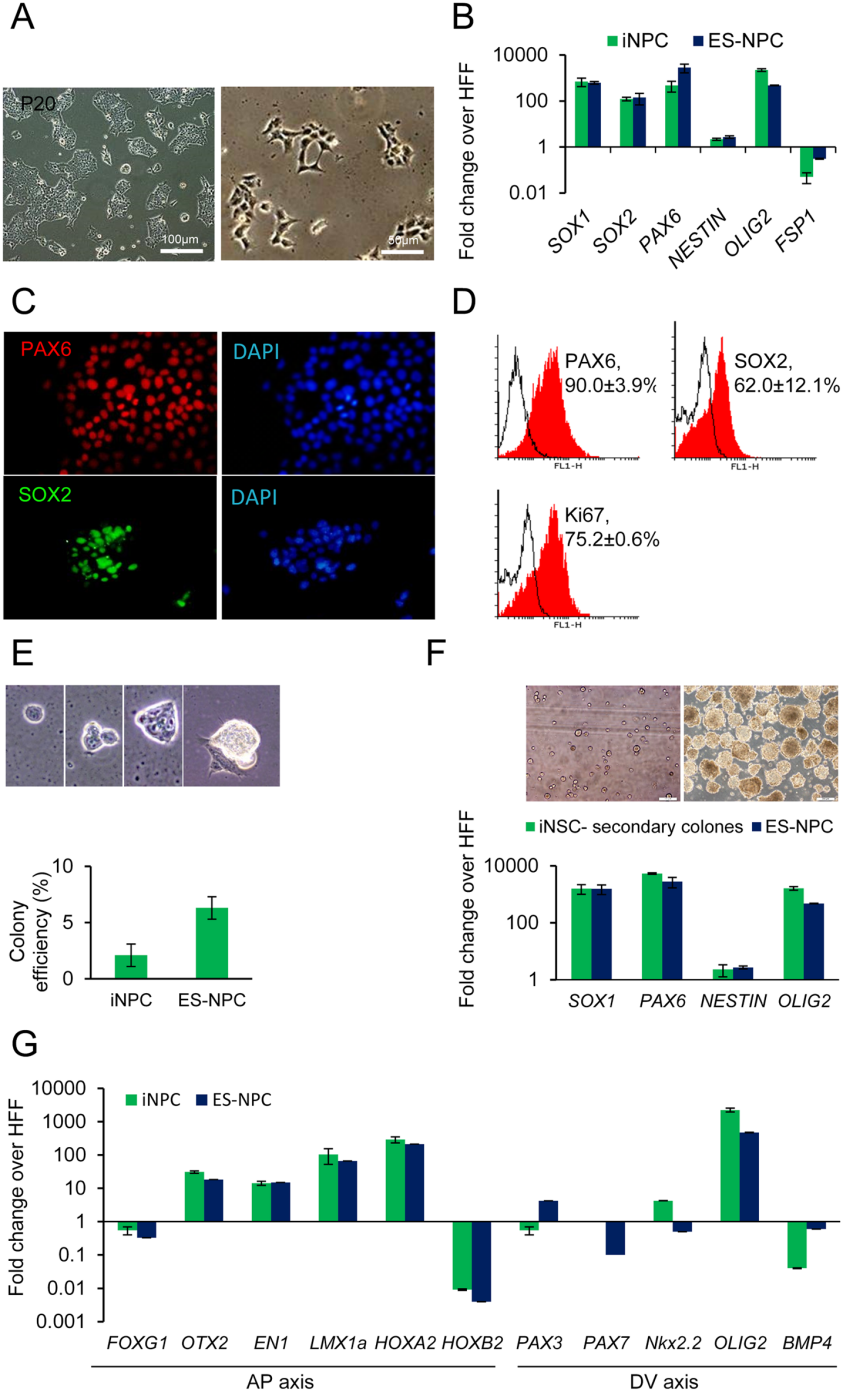
Characterization of established protein iNPCs. (A) Phase contrast of established iNSCs morphology at higher passages (p20). (B) Real-time analysis of iNPC and ES-derived NPC (ES-NPC) for neural progenitor markers. (C) Expressions of Nestin, PAX6 and SOX2 were visualized by immunostaining. Nuclei were counterstained with DAPI. (D) Flow cytometric analysis of neural progenitor markers (PAX6 and SOX2) and proliferation marker Ki67 in iNPCs. (E) Single-cell-per-well experiments illustrated that the established protein iNPC grew clonally. (F) Real-time analysis of secondary spheres of iNPCs for neural progenitor markers. (G) Relative expression levels of region-specific marker genes over human fibroblasts, whose expression was considered to be 1 for all genes.

For further characterization, we expanded the selected colonies for over 20 passages in M2 expansion medium. The iNPCs were highly proliferative, grew in colonies (Fig 3A), could be passed every 5–6 days, had the capability to be expanded to large numbers of cells and be cryopreserved. qRT-PCR confirmed that these iNPCs expressed a set of NPC marker genes that included *SOX1*, *SOX2*, *PAX6*, *NESTIN*, and *OLIG2* which was similar to the ES-NPCs (Fig 3B). Additionally, the expression level of fibroblast specific protein-1 (*FSP1*) significantly decreased in iNPCs compared with their starting cells (Fig 3B). The majority of cells were positive for PAX6, SOX2, and Ki67, as further confirmed by flow cytometric analysis (Fig 3C and 3D).

The first emerged colonies portrayed similar but not identical characteristics to ES-NPCs. However, after several passages they gradually became more similar to ES-NPC in terms of morphology and gene expression.

To further address full stem cell potential of these iNPCs, we initially examined their clonality by culturing single iNPCs and tracking them to determine their potential to proliferate into clonal neurospheres. The number of colonies was determined by counting all colonies within 14 days. We found that 2.1 ± 0.3% of the total seeded single cells formed a colony within 7 days (Fig 3E). Over the following days, these colonies grew rapidly and formed a large primary colony. However, the ability of our iNPC to form colonies from single cells was lower than those formed from the ES-NPCs (Fig 3E). When we dissociated these primary colonies into single cells, the extreme stress caused the vast majority of these cells to die. Therefore, we aimed to propagate iNPCs by passaging them at a low density in neurosphere culture in a serum-free suspension culture (Fig 3F). We reseeded the primary colonies into a single cell per well. They gave rise to secondary colonies, which we propagated at a low density in a 3D sphere culture. We demonstrated that these secondary derived cells expressed NPC related genes (Fig 3F). At higher densities, the adherent iNPCs had a tendency to aggregate spontaneously and detach from the plate to form floating neurospheres (data not shown). These cells also grew in size in the 3D sphere culture and formed secondary spheres upon dissociation.

To determine the regional identity of these iNPCs, we examined expression of the anterior-posterior and dorsal-ventral markers of the brain (Fig 3G). Real-time PCR assays showed that the expression of *FOXG1*, a forebrain restricted gene, was down regulated which suggested that iNPCs acquired a more posterior identity [5,33]. Interestingly, the iNPCs expressed the forebrain/midbrain gene *OTX2* and other midbrain genes *EN1*, and *LMX1A*. We also detected a strong expression of anterior hindbrain *HOXA2* but no expression of *HOXB2*, or posterior brain marker *HOXA4* in these cells (data not show). Notably, we detected strong expression of the ventral marker *OLIG2* and slight *NKX2*.*2* expression, whereas dorsal marker *PAX7* was undetectable; other dorsal markers such as *PAX3* and *BMP4* expressions downregulated in iNPCs. These observations together suggested an imaginary mid/hindbrain regional identity with a more ventral position for these iNPCs (Fig 3G).

Conjunctively, these data provided further evidence for the induction of a neural conversion program under our genetic material-free protocol.

### *In vitro* differentiation

**Fig 4:**
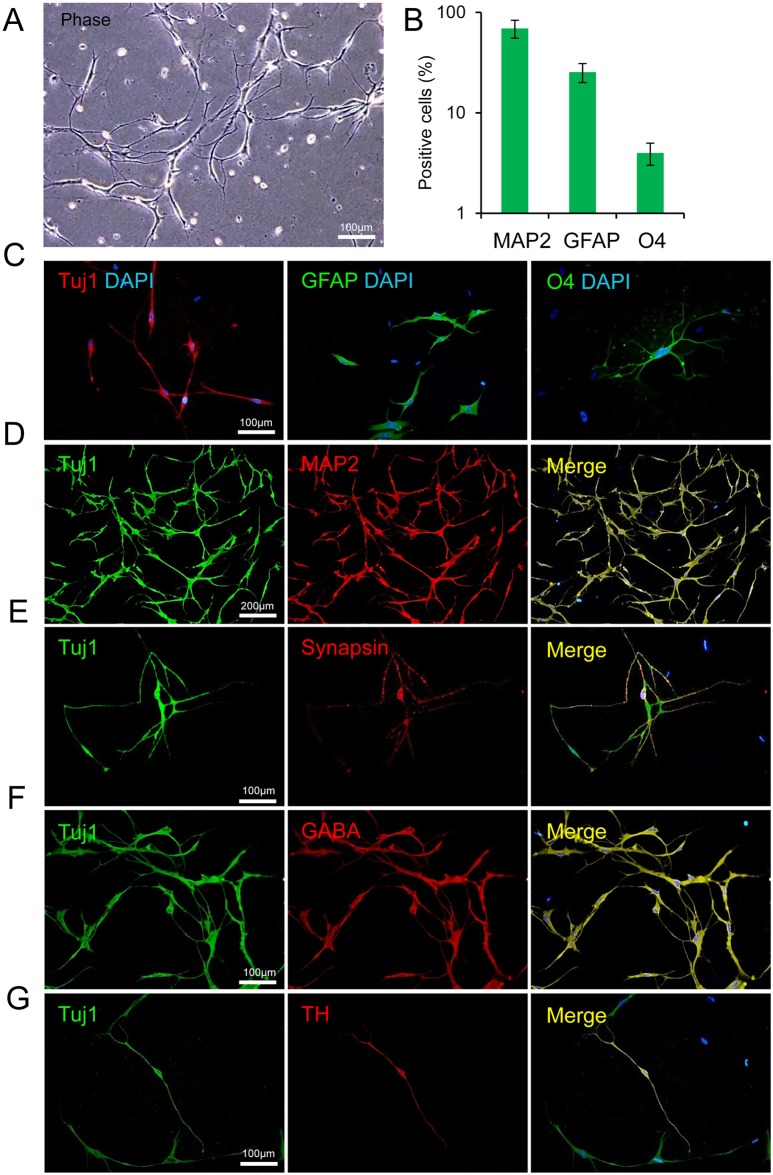
*In Vitro* differentiation potential of protein iNPCs. (A) Human protein iNPC-derived neural cells exhibited typical neuronal morphology. (B) Quantification of MAP2^+^ neurons, GFAP^+^ astrocytes and O4^+^ oligodendrocytes among total cells. Data are represented as mean ± SD. (C) Differentiation potential of iNSCs into neurons by TUJ1, astrocytes as determined by GFAP, and oligodendrocytes by O4 antibody via immunocytochemistry. DAPI staining is shown in blue. (D) MAP2^+^ neurons derived from iNPCs. (E) Dotted Synapsin expression in proximity of the TUJ1^+^ nerve fibers suggested morphological synapse formation. (F) The major population of iNPC-derived neurons was GABA^+^. (G) A small population of neurons were TH^+^. TUJ: β-Tubulin class III; MAP2: Microtubule-associated protein 2; TH: Tyrosine hydroxylase.

To further characterize iNPCs, we examined their differentiation potential by dissociating them into single cells which were cultured on laminin coated plates in the presence of BDNF, GDNF (10 ng/ml) and ascorbic acid (200 μM), and removal of growth factors from the NPC medium for up to 3–4 weeks. Under these conditions, cells spontaneously differentiated into a distinct neuronal morphology with elongated cytoplasmic process and multiple arborizing dendrites-like processes (Fig 4A). They had the capacity to differentiate into immature (TUJ1-positive cells) and mature neurons (MAP2-positive cells; Fig 4B–4D). GFAP positive astrocytes and O4 expressing oligodendrocytes could be also derived from piNPCs (Fig 4B and 4C). Upon further cultivation, the iNPC-derived mature neurons could express Synapsin-a marker for synapse formation *in vitro* (Fig 4E). In addition, different subtypes of neurons which included GABAergic (GABA positive cells) and dopaminergic (TH positive cells) were observed in the culture (Fig 4F and 4G). The major population of iNPC-derived neurons was GABA positive and a small population of neurons was TH positive. Together, these results suggested that our SOX2/SM treatment was sufficient to create a NPC-like population that had neurogenic/gliogenic potential.

### *In vivo* differentiation

**Fig 5:**
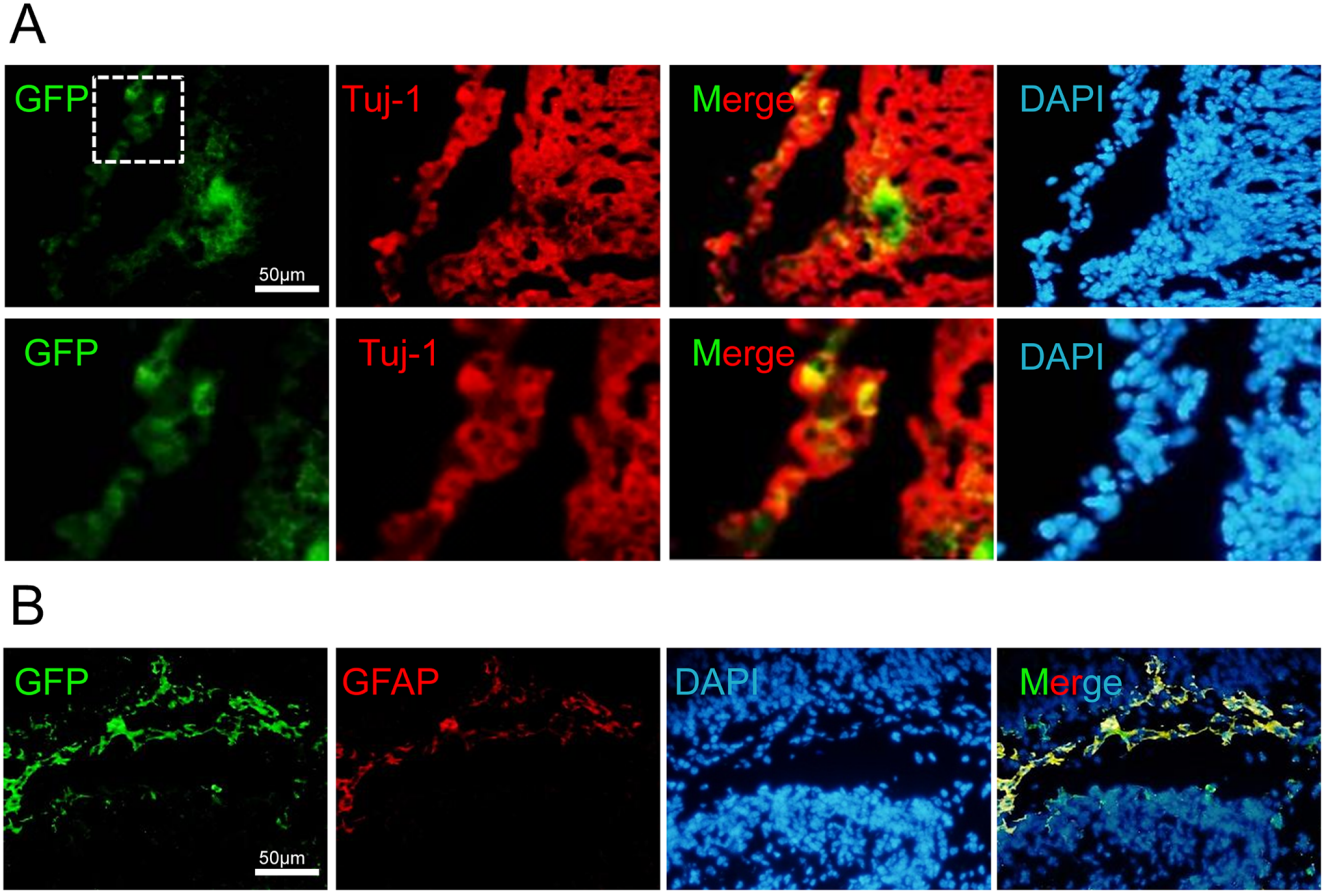
*In Vivo* transplantation of iNPCs. (A) Transplanted GFP^+^ protein iNPCs were TUJ^+^ at 10 days post-transplantation into the brain of a rat pup. Nuclei were counterstained with DAPI. (B) GFAP^+^ astrocytes were derived from GFP^+^ iNPCs *in vivo*.

Finally, we investigated the *in vivo* differentiation potential of iNPCs. We microinjected 10^5^ GFP labeled iNPCs into the brains of neonatal rat pups. After 10 days, we analyzed the fate of the transplanted cells in brain slices by staining against neural marker TUJ1 and a human-specific GFAP antibody, which indicated the cells had differentiated into astrocytes (Fig 5A and 5B). This study showed that iNPCs had the capability to generate differentiated neural cells (neurons and astrocytes) *in vivo*.

## Discussion

In this study we developed a protocol to generate genetic material-free iNPCs directly from human fibroblasts. This was achieved by SOX2 protein transduction in combination with a cocktail of SM in a 3D sphere culture.

Over the past few years numerous studies reported the direct generation of neural stem/progenitor cells from mouse and human fibroblasts by either the viral delivery of reprogrammed transcription factors in a transient manner [5,35] or of one or a mixture of more than 12 neural stem cell associated factors [8,9]. However, some employed human samples and the majority of methods have been based on integrating viruses [9,33]. Therefore, the risks for potential spontaneous reactivation of the viral transgenes such as gene disruption, instability and tumor formation limit their use in human therapy [36]. In contrast, in this study we utilized TAT fusion protein transduction and small chemicals to induce NPCs from human fibroblasts to eliminate these risks.

There are some other genetic material-free approaches that have generated iNPCs such as induction by forced growth of fibroblasts into a 3D sphere culture [29] or recently by only SMs and hypoxia [28]. To date, apart from a small study conducted on mouse fibroblasts by these conditions [28,29], there has been very little empirical research that report human gene-free iNPC generation. These protocols have not been applied to human fibroblasts which are of clinical interest and therefore currently not directly applicable to human systems. The current study has shown that either induction of hypoxia under 3D sphere culture [33], SM treatment or SOX2 protein alone did not convert human fibroblasts into iNPCs. We found that although the resultant cells expressed neuronal markers in the early stage of their conversion there was no colony formation after further culturing. These observations suggested that the cells underwent an incomplete conversion and appeared to have undergone substantial reprogramming to become neural stem/progenitor cell-like.These data also confirmed the hypothesis that unlike mouse cells, human cells needed more inductive signals and time to reprogram [37,38]. In this line, Mitchell et al. recently reported that human adult fibroblasts failed to convert into neural progenitors by viral transduction of *SOX2* alone despite upregulation of neural progenitor related genes [39]. Lu et al. reported similar results for generation of iNPCs from human adult fibroblasts by using SM alone [15]. Accordingly, we developed a modified method that could provide a supportive condition for induction and maintenance of fully converted NPC-like colonies. Previous studies have shown that an SM cocktail can support and sustain long-term self-renewal of human ESC-derived neural stem cells [34], generation of iNPCs [15,28] and enhance neural conversion efficiency [27]. Thus, we first provided a lineage specification reprogramming signal by 3D sphere culture, then protein transduction in the induction medium that contained the SMs which have been shown to promote NPC maintenance, SOX2 expression and facilitate neural conversion efficiency. Importantly, in this culture system NPC-like colonies emerged from human fibroblasts. Although, iNPC have been generated from human fibroblasts via non-integrating Sendai viruses in the presence of an SM cocktail similar to the one used in this study [15], the existence of the remnant viral genome of reprogramming factors within cells remains a safety concern. We have used SOX2 alone instead of four Yamanaka factors, which is degradable and a safer step. Considering that PSCs and other cell lineages might be induced during this process, we further analyzed these colonies for the expressions of pluripotent and non-neural lineage genes. The lack of these markers under our induction medium and spheroid differentiation condition excluded the possibility of pluripotent state induction and its rapid differentiation into NPCs. Therefore, we concluded that these colonies behaved more like the ES derived NPCs than pluripotent cells. This was also evident by the elevated expressions of NPC marker genes *SOX1*, *SOX2*, *PAX6*, *NESTIN*, and *OLIG2*, and down regulation of *FSP1*, which differed from PSCs. They could also be expanded to large numbers in the presence of a cocktail of growth factors and SM. Furthermore, we have suggest that this conversion is a gradual process in which the morphology and transcriptional program becomes more NPC-like over a period of time. The established iNPCs expressed typical NPC markers, could self-renew over several passages, grow clonally, generate neurospheres and were tripotent *in vitro*. In addition, the chemical cocktail in our culture system has kicked cells to acquire a mid/hindbrain-like fate. Interestingly, alternative chemical cocktails exhibited dorsal hindbrain [15] and ventral fore/midbrain regionalization [28,34]. These differences might be attributed to the different combination we used and dose-dependent effect of SMs on caudalizing and dorsalizing the iNPCs [40]. Of note, the high expression of *LMX1a* and the large number of differentiated GABA positive neurons generated from our iNPC suggested a bias toward GABAergic progenitors, which was supported by a past report that Lmx1a positive cells are predominantly GABAergic progeitors than dopaminergic *in vitro* [41].

In conclusion, the simple and facile system described here lays the foundation for future potential clinical applications which can alleviate concerns of the potential risks associated with conventional strategies. Beyond providing a safer means for the generation of patient-specific neural cells directly from autologous fibroblasts as a promising cell source, this strategy may also be a potential method for *in vivo/in situ* reprogramming of endogenous cells. Underlying many of these perspectives is the concept of human induced neuro-regeneration and reconstruction treatment options. However, our study is far from exhaustive and future investigations with further characterization such as electrophysiological characterization shall be considered. Moreover evaluation of cell survival, migration and differentiation as well as safety issue for longer time frame, may be very helpful to validate cell properties *in vivo*. Alternatively, it can be used in one of the neurological disease models to evaluate cell-based therapeutic effects. Further developments in lineage conversion approaches are poised to have substantial clinical implications for personalized cell therapy.

## Supporting Information

S1 Fig SDS-PAGE analysis of SOX2 and EGFP expression. Recombinant TAT-his-SOX2 and TAT-his-EGFP expressed and purified successfully. The purified proteins showed expected size band. The protein band was excised and analyzed using mass spectrometry that resulted in identification of related transcription factors as single protein in band.
(TIF)

S2 Fig Assessment of TAT-recombinant protein transduction. (A) Visualization of fusion proteins in monolayer cultured HFFs transduced with 10 μg/ml TAT-EGFP for 24 h. (B) Visualization of fusion proteins in a section of HFFs-sphere cultured with 10 μg/ml TAT-EGFP (24 h). Scale bar represent 50 μM in B. (C) TAT fusion protein were visualized by TAT staining after 48 and 72 h post TAT-SOX2 transduction.
(TIF)

S3 Fig Protocol design. (A) Schematic design of the protocol setting. Relative expression of neural genes with/without TAT-SOX2 protein transduction (+/- SOX2) and small molecules treatment (+/- SM). The results from three independent experiments are shown as mean±SD. (B) Morphological changes of different groups. Scale bars represent 200 μM.
(TIF)

S4 Fig Immunocytochemistry of NPC marker genes in HFFs. (TIF)

S1 Table Primers used for Real-time PCR. (DOC)

S2 Table Antibodies, sources, and dilutions. (DOC)
