# Supplementary figures and images for: Induction of Neural Progenitor-Like Cells from Human Fibroblasts via a Genetic Material-Free Approach
Source: PLoS One. 2015 Aug 12;10(8):e0135479. doi: 10.1371/journal.pone.0135479 (PMC4534403; doi:10.1371/journal.pone.0135479)

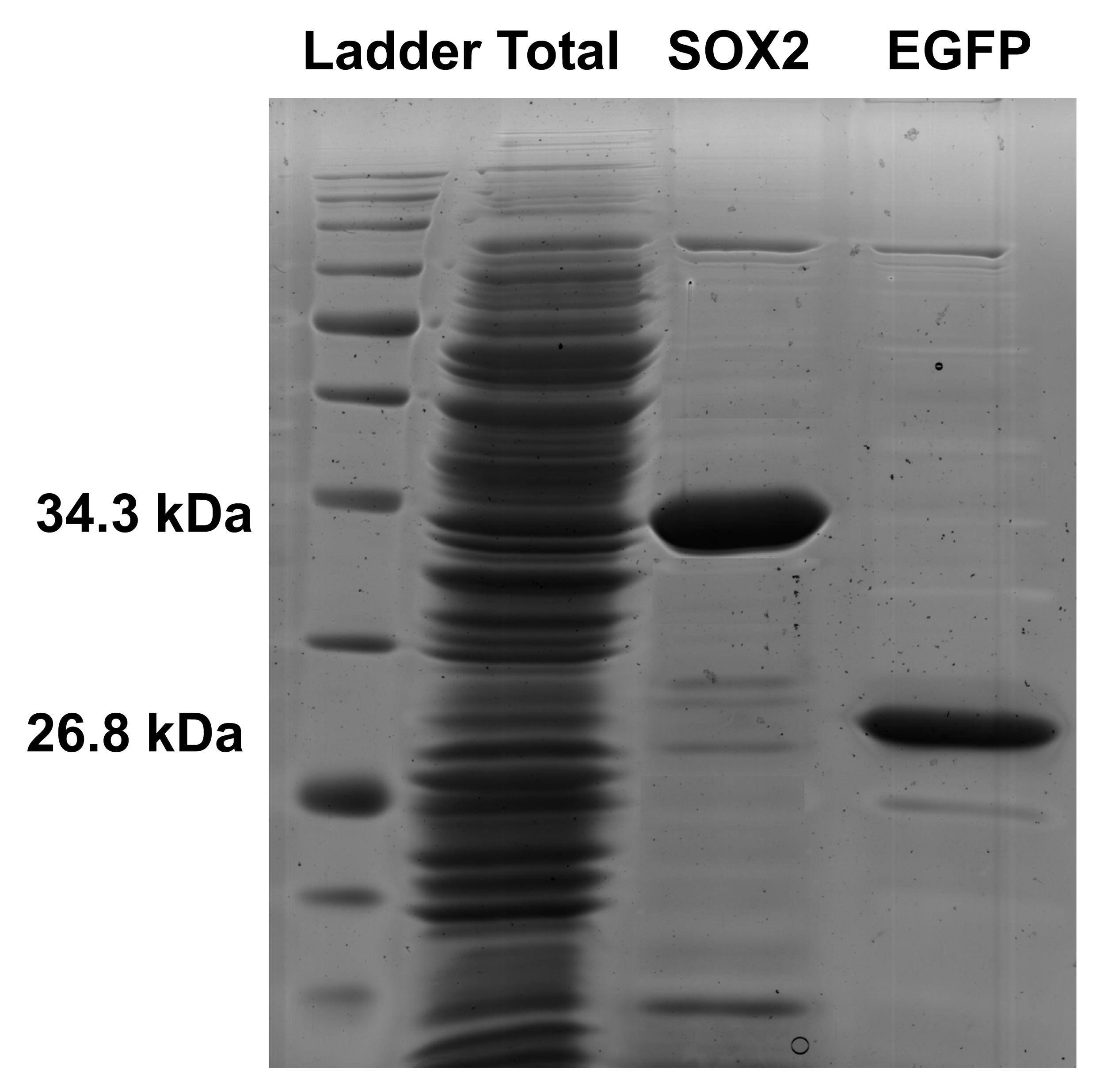

Supplement: S1 Fig — Recombinant TAT-his-SOX2 and TAT-his-EGFP expressed and purified successfully. The purified proteins showed expected size band. The protein band was excised and analyzed using mass spectrometry that resulted in identification of related transcription factors as single protein in band. (TIF) [file pone.0135479.s001.tif]

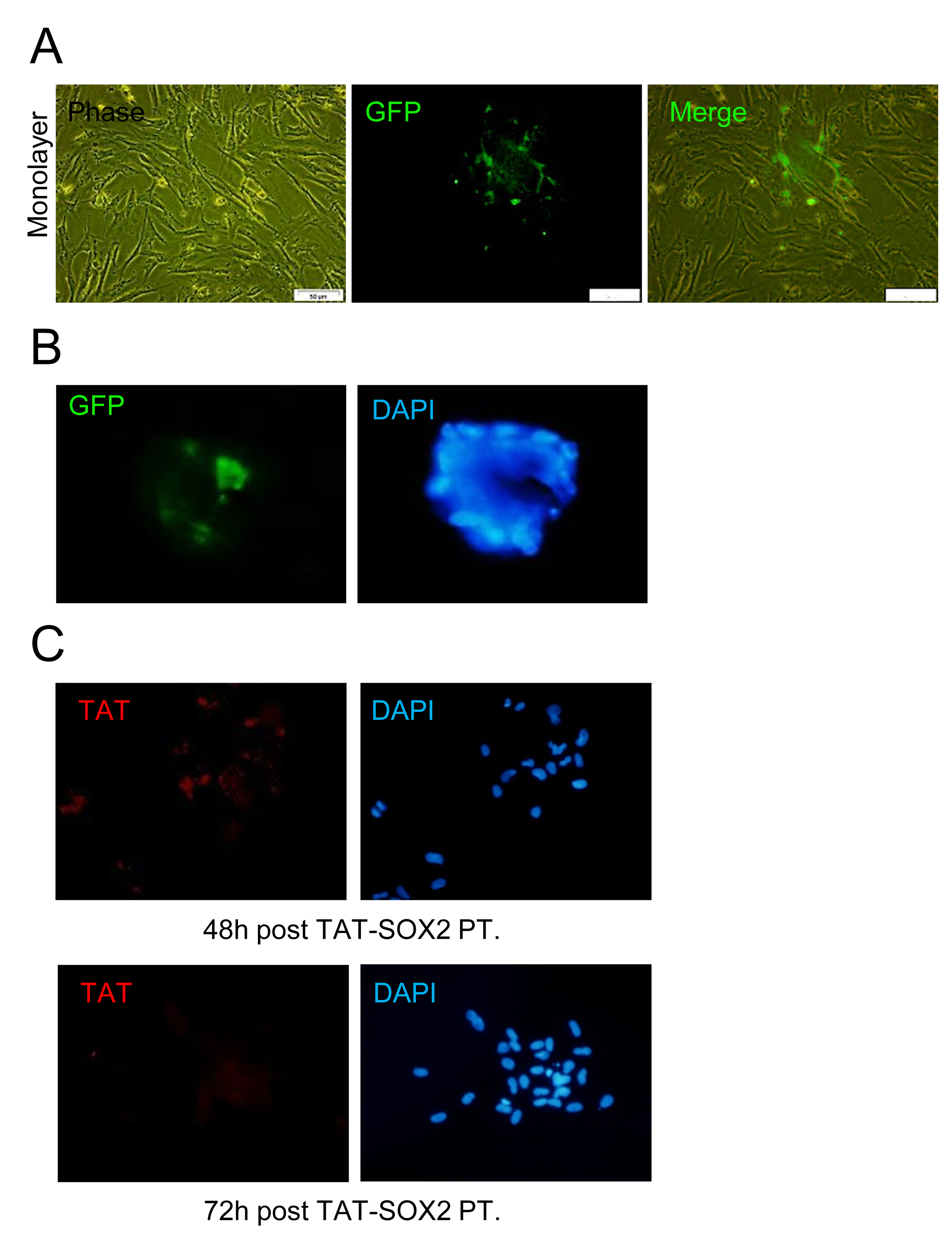

Supplement: S2 Fig — (A) Visualization of fusion proteins in monolayer cultured HFFs transduced with 10 μg/ml TAT-EGFP for 24 h. (B) Visualization of fusion proteins in a section of HFFs-sphere cultured with 10 μg/ml TAT-EGFP (24 h). Scale bar represent 50 μM in B. (C) TAT fusion protein were visualized by TAT staining after 48 and 72 h post TAT-SOX2 transduction. (TIF) [file pone.0135479.s002.tif]

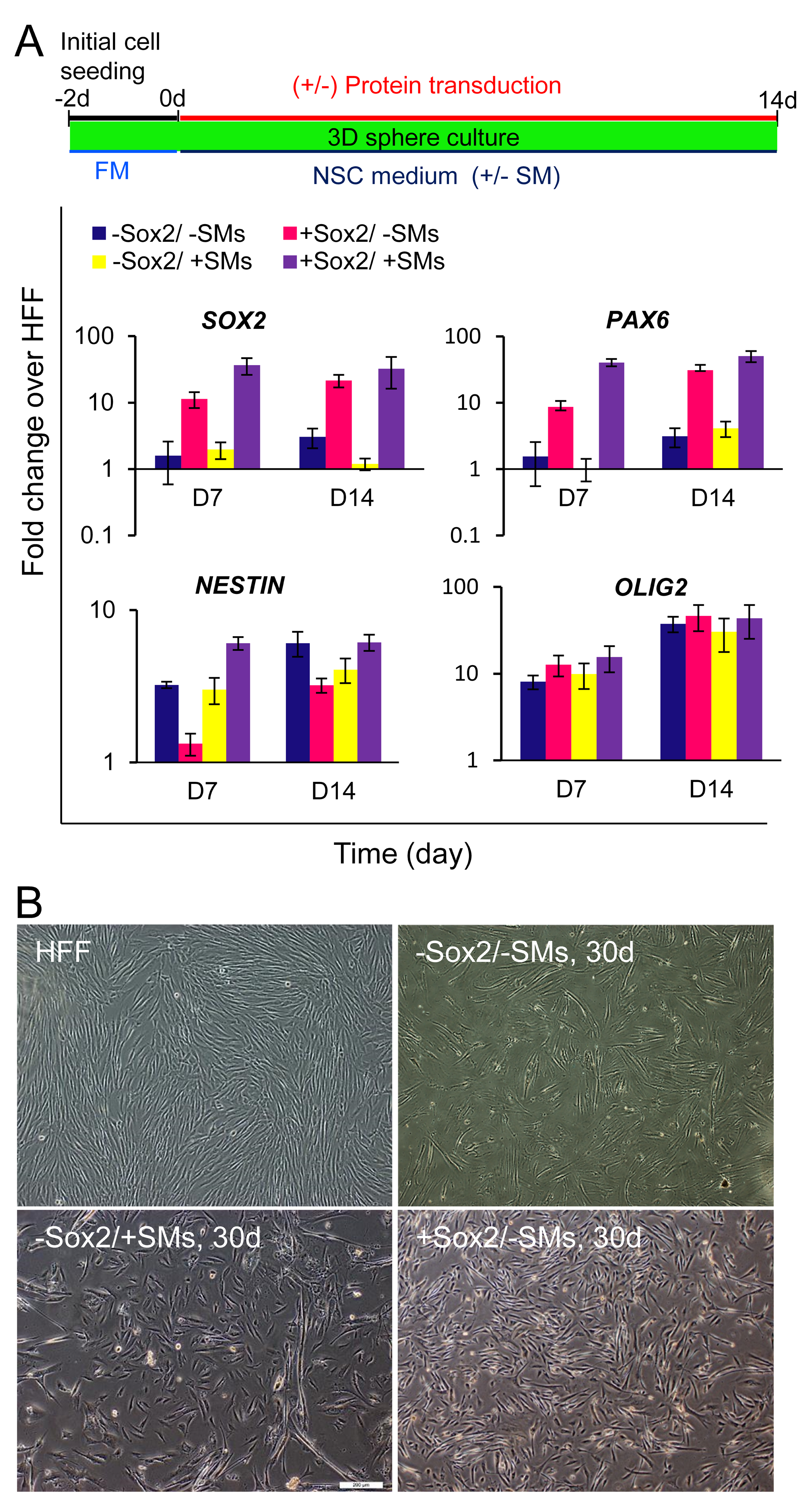

Supplement: S3 Fig — (A) Schematic design of the protocol setting. Relative expression of neural genes with/without TAT-SOX2 protein transduction (+/- SOX2) and small molecules treatment (+/- SM). The results from three independent experiments are shown as mean±SD. (B) Morphological changes of different groups. Scale bars represent 200 μM. (TIF) [file pone.0135479.s003.tif]

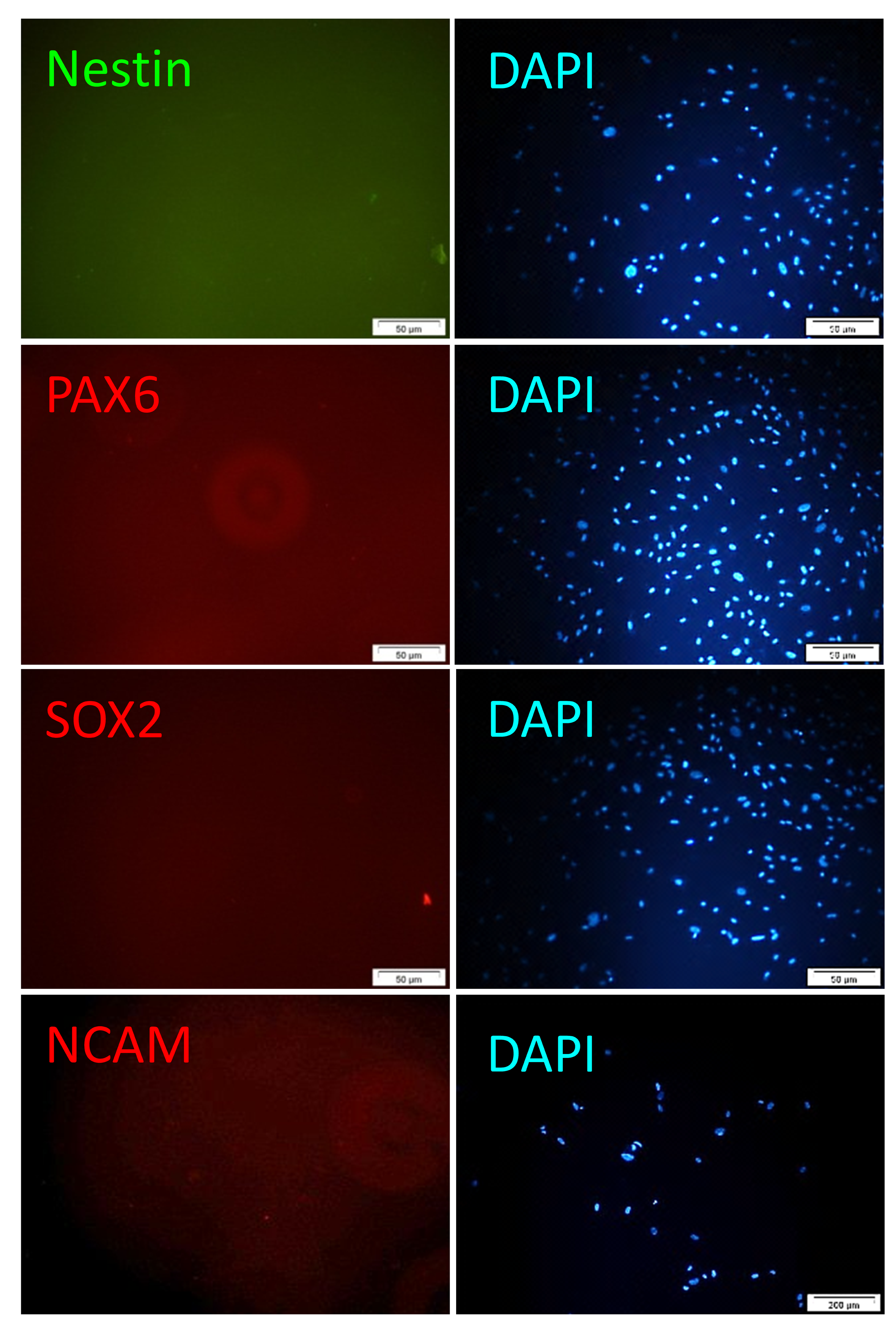

Supplement: S4 Fig — (TIF) [file pone.0135479.s004.tif]
