# Supplementary material for: Induction of Neural Progenitor-Like Cells from Human Fibroblasts via a Genetic Material-Free Approach
Source: PLoS One. 2015 Aug 12;10(8):e0135479. doi: 10.1371/journal.pone.0135479 (PMC4534403; doi:10.1371/journal.pone.0135479)
Supplement: S1 Table — (DOC) [file pone.0135479.s005.doc]

**S1 Table. Primers used for Real-time PCR.**

| ***Primer name*** | **Primer sequences** | ***Primer name*** | **Primer sequences** | |  |
| --- | --- | --- | --- | --- | --- |
| *SOX1* | F: 5' TTT TTG GAT CCA TTC TTT CTC TCA A3'  R: 5' ATC CAC TCG GTG CTT TTG TG3' | *NKX2.2* | F: 5' CGCTATTGTCAGCCGTCTTCTAA 3' R: 5' TGCCAGTTGTCAGAA 3' | |  |
| *SOX2* | F: 5' GGA GTG CAA TAG GGC GGA AT3'  R: 5' CCA GTT GTA GAC ACG CAC CT 3' | *PAX3* | F: 5' CGAACACGTTCGACAAAAGCA 3'  R: 5'GCACACAAGCAAATGGAATG 3' | |  |
| *PAX6* | F: 5' GTC CAT CTT TGC TTG GGA AA 3'  R: 5' TAG CCA GGT TGC GAA GAA CT 3' | *PAX7* | F:5'AGAGGAGGAGGATGAAGC 3' R:5'TATGTCTGGGTAGTAGTCCTCCT 3' | |  |
| *NESTIN* | F: 5' CTC CAG AAA CTC AAG CAC C 3'  R: 5' TCC TGA TTC TCC TCT TCC A 3' | *HOXA2* | F: 5' AGGAGGACGAGGAAGAGA 3'  R: 5' ACTGGGAAACTTTGGGAG 3' | |  |
| *OLIG2* | F: 5' CGA CTC ATC TTT CCT CCT TCT CTA A3' R: 5' CGC ACT TAC CTC ATC ATT G3' | *HOXB2* | F: 5' GCCACGTCTCCTTCTC 3'  R: 5' CTTCTCCAGTTCCAGCAG 3' | |  |
| *FSP1* | F: 5' ACT TGG ACA GCA ACA GGG AC 3'  R: 5' CCC CAA CCA CAT CAG AGG AG 3' | *EN1* | F: 5' CGCAGCAGCCTCTCGTATGG 3‘  R: 5' GCCGCTTGTCCTCCTTCTTCG 3' |  | |
| *NANOG* | F: 5' CAG CTA CAA ACA GGT GAA GAC 3'  R: 5' TGG TGG TAG GAA GAG TAA AGG3' | *FOXG1* | F: 5' CCGCAGTGATCAACAAAGAC 3'  R:5' CTCTTTGGGCTTCTCGTCTG 3' |  | |
| *OCT4* | F:5' TCT ATT TGG GAA GGT ATT CAG C3'  R:5' ATT GTT GTC AGC TTC CTC CA 3' | *LMX1a* | F: 5' GCCTCATTTGAAGTATCCTCC 3'  R: 5'GCTTCTTCATCTTCGCTCTC 3' |  | |
| *REX1* | F:5' TTTACGTTTGGGAGGAGG 3’  R:5' GTGGTCAGCTATTCAGGAG3’ | *BMI1* | F: 5' GTGTGCTTTGTGGAGGG3'  R: 5'AGTAGTGGTCTGGTCTTGTG3' |  | |
| *T* | F: 5' AATGGTCCAGCCTTGGAAT 3'  R: 5‘CGTTGCTCACAGACCACA 3' | *BMP4* | F: 5' GGCCAGCATGTCAGGATTAG3' R: 5'CACATCGCTGAAGTCCACAT3' |  | |
| *GATA4* | F: 5' CCT GTC ATC TCA CTA CGG 3'  R:5'GCT GTT CCA AGA GTC CTG 3' |  |  |  | |
| *SOX17* | F: 5'CTCTGCCTCCTCCACGAA 3'  R: 5' CAGAATCCAGACCTGCACAA 3' |  |  |  | |
| *CXCR4* | F: 5' AACTTCAGTTTGTTGGCTGC 3'  R: 5'CATTTCCTCGGTGTAGTTATCTG 3' |  |  |  | |
| *GAPDH* | F: 5' CTC ATT TCC TGG TAT GAC AAC GA 3'  R:5' CTT CCT CTT CTC CTC TTG CT 3' |  |  |  | |
| *OTX2* | F: 5' CTCTGAACCTGTCCA 3' R: 5' AGCAAGTCCATACCCGAA 3' |  |  |  | |
