# Supplementary material for: Induction of Neural Progenitor-Like Cells from Human Fibroblasts via a Genetic Material-Free Approach
Source: PLoS One. 2015 Aug 12;10(8):e0135479. doi: 10.1371/journal.pone.0135479 (PMC4534403; doi:10.1371/journal.pone.0135479)
Supplement: S2 Table — (DOC) [file pone.0135479.s006.doc]

**S2 Table. Antibodies, sources, and dilutions.**

| **Antibody** | **Source** | **dilution** |
| --- | --- | --- |
| TAT | Cell aplication | 1/200 |
| Sox2 | Santa Cruz | 1/100 |
| Ncam | Santa Cruz | 1/200 |
| Sox1 | abcam | 1/200 |
| Pax6 | Santa Cruz | 1/200 |
| Nestin | Santa Cruz | 1/200 |
| Ki67 | abcam | 1/200 |
| Tuj1 | Sigma-Aldrich | 1/400 |
| Tuj1 | abcam | 1/300 |
| MAP2 | Sigma-Aldrich | 1/200 |
| GFAP | Biorbyt | 1/200 |
| O4 | Chemicon | 1/200 |
| TH | Novus biologica | 1/200 |
| TH | Sigma-Aldrich | 1/200 |
| GABA | Chemicon | 1/200 |
| Synapsin | Sigma-Aldrich | 1/200 |
| Goat anti mouse Alexa 488 | Invitrogen | 1/500 |
| Goat anti mouse Alexa 568 | Invitrogen | 1/500 |
| Goat anti rabbit Alexa 568 | Invitrogen | 1/500 |
| Goat anti rabbit Alexa 546 | Invitrogen | 1/500 |
| Goat antimouse IgM 488 | Invitrogen | 1/500 |
